# Infection control-termendous need for data

**DOI:** 10.1186/1753-6561-5-S6-P241

**Published:** 2011-06-29

**Authors:** M Schnürer, F Oemig, D Colaert

**Affiliations:** 1 AGFA Healthcare, Bonn, Germany; 2 AGFA Healthcare, Gent, Belgium

## Introduction / objectives

Real-time high-quality data is a vital requisite to control HAI (Hospital Aquired Infections). Early identification of epidemics’ spreading dynamics is of prime importance to locate the source of HAI and take measures in hospitals to a national level. Yet, technical possibilities to influence necessary key indicators and putting authorities in an acting rather than reacting position are not entirely used. Today’s proceedings use notification by fax with receiving instistutions passing them on to RKI (Robert Koch Institut) for further research and publishing. This results in low notification rates, data privacy issues, slow communication and few data mining possibilities. Authorities cope with incomplete forms and manual transcription into various systems.

## Methods

A thorough analysis by research of literature and implementation guidelines has revealed a set of options, how to improve the process.

## Results

The designed solution is adaptable to stakeholders’ IT-systems, increases data quality and quantity, the availability for stakeholders and empowers them to further exploit the data. Hence, secure web-services are used for transport, CDA for structured data encoding, Arden Syntax or OWL as a knowledge representation language combined with a dashboard for visualization and central data repository. As a result the time span between first detection and reaction reduces significantly. Reports increase awareness and ensure visibility of epidemic situations as well as allowing electronic surveillance or scientific analysis.

## Conclusion

Handling notifiable diseases remains behind technical means. The proposed solution solves identified problems offering a flexible, reliable and timeliness possibility. A first pilot shows that pathogens of different Laboratories are traceable and exploitable in a Population Dashboard.

## Disclosure of interest

None declared.

